# A simplified multiplex methylated DNA testing for early detection of colorectal cancer in stool DNA

**DOI:** 10.1186/s12876-022-02512-6

**Published:** 2022-10-06

**Authors:** Yanmiao Dai, Guodong Zhao, Jun Yang, Xilang Zhou, Shangmin Xiong, Xirong Lu, Liming Gao, Jianfang Wu, Zouhua Xu, Sujuan Fei, Minxue Zheng, Hongwei Xu

**Affiliations:** 1 Department of Spleen and Stomach Diseases, Kunshan Hospital of Traditional Chinese Medicine, Kunshan, 215300 Jiangsu China; 2 Department of Gastroenterology, Affiliated Hospital of Xuzhou Medical University, Xuzhou, 221002 Jiangsu China; 3 Zhejiang University Kunshan Biotechnology Laboratory, Zhejiang University Kunshan Innovation Institute, Kunshan, 215300 Jiangsu China; 4 Suzhou VersaBio Technologies Co. Ltd., Kunshan, 215300 Jiangsu China; 5 Suzhou Institute of Biomedical Engineering and Technology, Chinese Academy of Sciences, 88 Keling Road, SND, Suzhou, 215163 Jiangsu China

**Keywords:** Colorectal cancer, Early detection, Stool, Simplified, DNA methylation

## Abstract

**Background:**

ColoDefense1.0 assay has demonstrated its excellent sensitivity and specificity for early detection of colorectal cancer (CRC) by detecting the methylation levels of *SDC2* and *SEPT9*, while exhibited limitations on relatively large sample capacity required and limited detection throughput by applying triplicate PCR reactions for each sample. In this study, ColoDefense1.0 was simplified and optimized into ColoDefense2.0 in a single PCR reaction.

**Methods:**

A total 529 stool specimens were collected, and 244 CRC patients, 34 patients with advanced adenomas (AA), 64 with small polyps (SP) and 187 control subjects were divided in training and validation cohorts. Methylation levels of *SEPT9* and *SDC2* were examined by qPCR reactions in triplicate or single.

**Results:**

The stool DNA quantity stored in preservative buffer at 37 °C up to 7 days exhibited no significant decrease. In the training cohort, when the number of replicates reduced from 3 to 1, the overall performance of ColoDefense2.0 was identical to that of ColoDefense1.0, showing sensitivities of 71.4% for AA and 90.8% for all stage CRC with a specificity of 92.9%. In the validation cohort, sensitivities of SP, AA and CRC using ColoDefense2.0 were 25.0%, 55.0% and 88.2%, increased from 14.1% (20.3%), 40.0% (40.0%) and 79.4% (67.6%) using *SDC2* (*SEPT9*) alone; along with an overall specificity of 90.2%, decreased from 94.1% (95.1%) using *SDC2* (*SEPT9*) alone.

**Conclusion:**

The simplified ColoDefense test maintained the overall performance while reduced the number of PCR reactions to 1/3, and provided an effective and convenient tool to detect early CRC and precancerous lesions and potentially improve the compliance of screening.

**Supplementary Information:**

The online version contains supplementary material available at 10.1186/s12876-022-02512-6.

## Introduction

Globally, colorectal cancer (CRC) is the third most commonly diagnosed cancer and the second leading cause of cancer death, with over 1.9 million new cases and 0.9 million death estimated to occur in 2020, contributing to 10.0% of new cancer cases and 9.4% of cancer deaths [1]. Participation in CRC screening and removal of adenomatous polyps can significantly reduce the mortality and incidence of CRC. For example, colonoscopy prevalence in the U.S. adults of 50 years or older has increased from 20 to 61% from 2000 to 2018, contributing to the significantly decreased incidence rate from approximately 185 to 115 per 100,000 population in the U.S [2]. However, such high colonoscopy prevalence is difficult to reach in developing countries or in the COVID-19 pandemic, due to insufficient availability in medical resources, limited awareness, invasiveness and bothersome bowel preparation [3]. In this light, a number of noninvasive methods as preliminary screening strategies have been developed, such as high-risk factor questionnaire, fecal occult blood test (FOBT), fecal immunochemical test (FIT), plasma or stool DNA tests, with the objectives to improve the compliance of screening and to screen out high-risk population for diagnosis and treatment [4–7]. Our team previously has developed a cell-free DNA (cfDNA) test, ColoDefense1.0, that detected methylated *SEPT9* and *SDC2* simultaneously in a multiplex qPCR assay, exhibiting outstanding sensitivities of 92.3% for all stage CRC and 66.7% for AA with a specificity of 93.2% in stool samples, and sensitivities of 88.9% for CRC and 47.8% for AA with a specificity of 92.8% in plasma samples [8, 9]. These results demonstrated the potential of ColoDefense1.0 to become an effective and accurate tool for early detection of CRC.

Due to trace amount of cfDNA in the stool or plasma samples in detecting CRC, it is a common practice to apply multiple PCR reactions in a test to improve the detection accuracy. Oh et al*.* reported a blood-based qPCR assay detecting *SDC2* methylation that was run in triplicate, and then a stool-based quantitative methylation-specific real time PCR (qMSP) method that implemented consecutive two rounds of PCR reactions to detect the methylation of *SDC2* [10, 11]. Epi proColon, the first blood-based assay approved by FDA on methylation of *SEPT9*, were run in triplicate and exhibited a sensitivity of 22% for AA, 68% for all stages of CRC [12]. This assay was then applied in a prospective study using triplicate PCR reactions, resulting in a sensitivity of 48.2% for all stage CRC [13]. Our previous study applied and compared 2/3 and 3/3 rules in detecting methylated SFRP2 and *SDC2* in stool to optimize the cut-off values, resulting in sensitivities of 61.5% for detecting AA and 88.5% for early stage CRC (stage 0-II) by 2/3 rule [14]. A triplicated PCR reaction was applied in ColoDefense1.0 as well. However, it is obvious that the detection throughput would be limited by multiple repetitions in PCR reactions. Therefore, this study aims to simplify and optimize ColoDefense1.0 assay in stool samples to a single PCR reaction.

## Materials and methods

### Sample collection

In this study, we enrolled 584 participants who underwent colonoscopy due to visited outpatient clinics or physical examination from two hospitals including two independent cohorts (Additional file 1: Fig. S1). The inclusion criteria consisted of the following: aged 18 or older, no history of CRC, nonpregnant, having colonoscopy results, and participants with abnormal colonoscopy results should have pathological diagnosis results. During stool sample collection, efforts were made to avoid transferring urine into the collection tube, and diarrhea samples were not collected. The exclusion criteria were as follows: other gastrointestinal malignancies, unable or unwilling to accept colonoscopy, missing or incomplete sample information, insufficient or excessive stool volume, repeated sampling, and insufficient DNA indicated by low *ACTB* levels (see Data Analysis). After excluding unqualified samples, training cohort collected at the Affiliated Hospital of Xuzhou Medical University included 142 CRC patients, 14 patients with advanced adenomas (AA, an adenoma with size ≥ 1.0 cm, significant villous features (> 25%) or high-grade dysplasia) and 85 control subjects who underwent colonoscopy for data analysis (Additional file 1: Fig. S1). The validation cohort included 102 CRC patients, 20 AA patients, 64 small polyp (SP, an adenoma < 10 mm in size without high-grade dysplasia and villous histologic features, or hyperplastic polyp < 10 mm in size) patients, and 102 control subjects collected at the Kunshan Hospital of Traditional Chinese Medicine (Additional file 1: Fig. S1). The control subjects enrolled in training and validation cohorts included diverticula, colitis and subjects with no evidence diseases. Training cohort was used for optimizing cut-off value, and the validation cohort was used for verifying the cut-off value, the colonoscopy and pathological results in validation cohort was blinded for laboratory staff. Stool samples (approximately 5 g) were collected before purgative bowel preparation or colonoscopy by using the single-use disposable buckets from Suzhou VersaBio Technologies Co., Ltd., and then each stool specimen was transferred into a 50 mL tube containing 25 mL of preservative buffer (Suzhou VersaBio Technologies Co., Ltd., Kunshan, China). All stool samples were stored at room temperature for no more than 7 days or at − 80 °C for longer-term storage before usage. This study was approved by the Institutional Review Board of the Affiliated Hospital of Xuzhou Medical University and Kunshan Hospital of Traditional Chinese Medicine, and the informed consent was obtained for all participating patients and control subjects.

### Stool DNA stability study

The aim of this procedure was to investigate the efficiency of preservative buffer during stool sample collection and transportation. Two stool samples were collected from each of 10 healthy donors, one sample collected in a tube containing 25 mL preservative buffer (PS) and the other in 25 mL deionized water (DS). Each sample was transfer to the laboratory within 2 h from collection. The human genomic DNA for PS were extracted immediately, and then the remaining PS and DS were stored at 37 °C (approximately the highest average temperature in summer). The DS were processed for human genomic DNA extraction after 2 h and the remaining PS were processed for human genomic DNA extraction at 1, 3 and 7 days. Next, all stool DNAs were bisulfite-treated and detected by *ACTB* qPCR reaction.

### The simplified assay

We named the simplified assay as “ColoDefense2.0”. Compared to the original assay (ColoDefense1.0), 3 PCR replicates were reduced to 1 PCR replicate in ColoDefense2.0, and the reaction volume was increased from 30 to 50 μL. The cut-off value for ColoDefense2.0 was set as follows: the stool sample was considered ‘invalid’ if the *ACTB* Ct value was greater than 43.0, the optimized cut-off values for methylated *SEPT9* and *SDC2* were Ct values less than 38.0 and 40.0, respectively (Additional file 2: Table S1), and a stool sample would be scored positive when any methylated marker was scored positive.

### DNA extraction, bisulfite treatment and methylation detection

Stool samples were homogenized and centrifugated, and 150 μL supernatants were transferred for human genomic DNA extraction by using a Versa-Autopure Nucleic Acid Purification System (Suzhou VersaBio Technologies Co., Ltd., Kunshan, China). All samples underwent lysis and two washing steps and were finally eluted in 100 μL of elution buffer. Bisulfite treatment of purified DNA were performed with a fast bisulfite conversion kit (Suzhou VersaBio Technologies Co., Ltd.) according to previous study [8]. The purification of the converted products also using the Versa-Autopure Nucleic Acid Purification System by three washing steps and were finally eluted in 100 μL of elution buffer.

The methylation detection of ColoDefense1.0 according to the published procedure [8], and ColoDefense2.0 test include 25 µL template and 25 µL PCR master mix. Both ColoDefense1.0 and ColoDefense2.0 were analyzed on LC480-II thermal cycler (Roche Diagnostics, Basel, Switzerland) using the following cycling conditions: activation at 95 °C for 30 min, 50 cycles of 95 °C for 10 s, 56 °C for 30 s, and final cooling to 40 °C for 30 s.

### Analytical performance of the simplified assay

To determine the limit of detection (LoD) for two methylated markers in ColoDefense2.0, fully methylated genomic DNA were diluted into unmethylated genomic DNA (1:100, w/w) to create mixtures. For *SDC2*, fully methylated genomic DNA with concentration gradient of 50 pg/reaction, 25 pg/ reaction and 12.5 pg/reaction tested, and each reaction was considered positive based on ColoDefense2.0 cut-off value. For *SEPT9*, fully methylated genomic DNA with concentration gradient of 200 pg/reaction, 100 pg/ reaction and 50 pg/reaction tested, and each reaction was considered positive based on ColoDefense2.0 cut-off value. Meanwhile, 20,000 pg/reaction fully unmethylated genomic DNA was used for analyzing the LOB (Limit of Blank) of the test. Each concentration sample was repeated for 20 times.

### Data analysis

For ColoDefense1.0, Mean Ct values for each methylated marker were used for plotting receiver operating characteristic (ROC) curves, and the Ct values for ColoDefense2.0 were used for plotting ROC curves. The confidence interval (CI) of an area under the curve (AUC) was calculated. For those subjects without amplification signals in qPCR reaction, their Ct values were set to 50 (the maximal number of PCR cycles).

## Results

A total of 529 participants were enrolled and their characteristics are shown in Table 1. Among all participants, 244 were CRC patients (including 8 of stage 0, 39 stage I, 72 stage II, 80 stage III, 23 stage IV and 22 of unknown stage), 98 patients having polyps (including 34 AA patients and 64 SPs), and 187 normal controls. Percentages of male patient, median and range of age at diagnosis for CRC patients was 56.3%, 63, 20–84 in the training cohort, comparable to those in the validation cohort (54.9%, 61, 27–89). However, the age distribution for control subjects was younger than that of the CRC patients in both training cohort and validation cohort (Additional file 2: Table S2).

**Table 1:** The characteristics of subjects enrolled in this study

Characteristics	Training cohort				Validation cohort		
	CRC (n = 142)	AA (n = 14)	Control (n = 85)	CRC (n = 102)	AA (n = 20)	SP (n = 64)	Control (n = 102)
Sex of patient							
Male	80 (56.3%)	11 (78.6%)	43 (50.6%)	56 (54.9%)	12 (60.0%)	35 (54.7%)	43 (42.2%)
Female	62 (43.7%)	3 (21.4%)	42 (49.4%)	46 (45.1%)	8 (40.0%)	29 (45.3%)	59 (57.8%)
Age at diagnosis (years)							
Median	63	65	51	61	58	60	45
Mean	61	63	49	61	57	57	46
Range	20–84	39–76	26–83	27–89	33–75	30–92	21–80
TNM stage		–	–		–	–	–
0	3			5			
I	19			20			
II	46			26			
III	41			39			
IV	11			12			
Unknown	22			-			

**Fig. 1:**
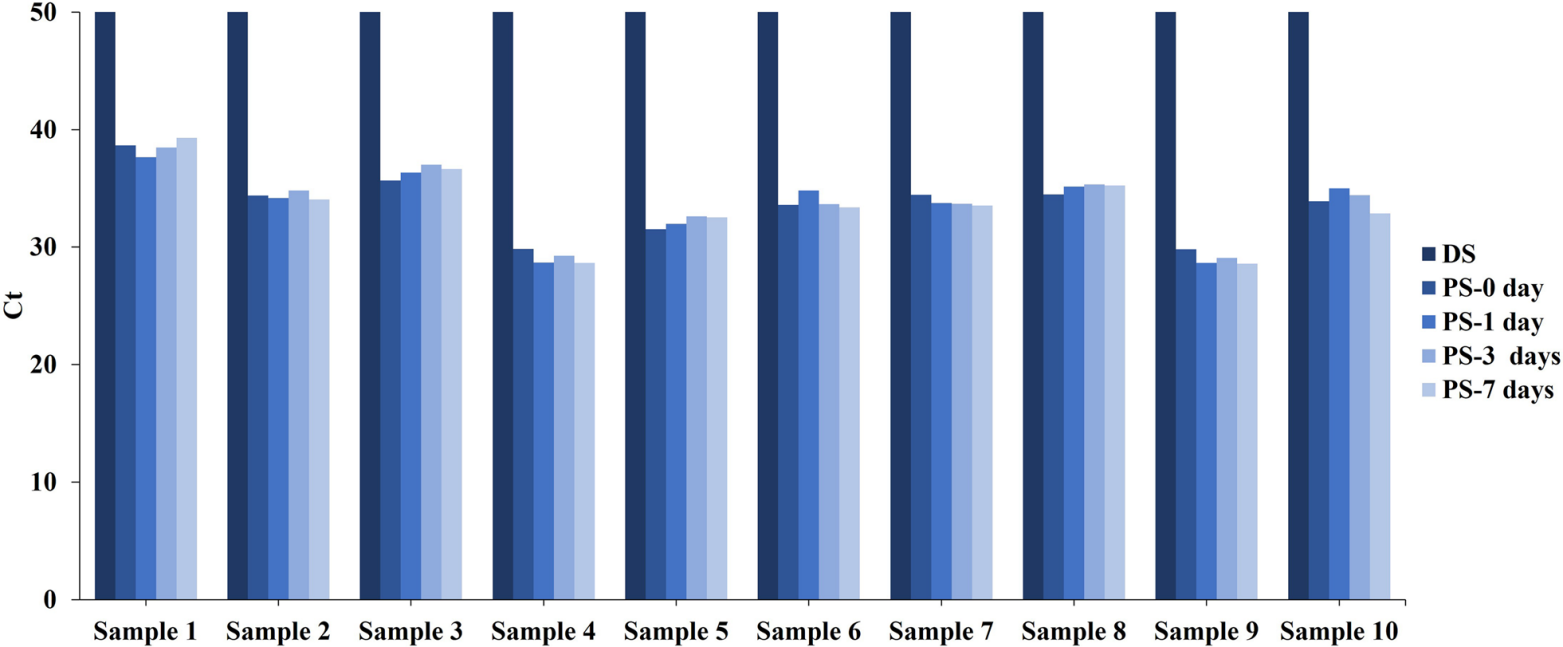
The effect of protect stool human genomic DNA with or without preservative buffer. Ct values of *ACTB* from 10 samples collected in deionized water (DS) were labeled in dark blue, those from samples with preservative buffer (PS) were labeled in lighter blue

Stool DNAs in samples collected without preservative buffer showed a drastic degradation after 2 h at 37 °C, and no amplification signal was detected in any samples, as shown in Fig. 1. From fresh (0 day) to 7 days, Ct values of *ACTB* from samples in preservative buffer exhibited no significant increase, which validated that no significant degradation of stool DNA and the stability of stool DNA was successfully preserved at 37 °C for at least a week.

To evaluate the limit of detection for methylated *SDC2* and *SEPT9* alone, methylated human genomic DNA with different concentrations were tested for 20 replicates. As shown in Table 2, ColoDefense2.0 was able to detected *SDC2* and *SEPT9* at a concentration of 25 and 100 pg/reaction at 100% positive rate, respectively. And neither methylated *SDC2* or *SEPT9* showed positive signals in fully unmethylated human genomic DNA.

**Table 2:** Limit of detection for two methylated markers in ColoDefense2.0

*SDC2*			*SEPT9*			*ACTB*		
CONC (pg/reaction)	PR (%)	Mean Ct	CONC (pg/reaction)	PR (%)	Mean Ct	CONC (pg/reaction)	PR rate (%)	Mean Ct
0	0	–	0	0	–	20,000	100	25.6 ± 0.1
50	100	35.8 ± 0.4	–	–	–	5000	100	27.3 ± 0.1
25	100	37.6 ± 0.9	–	–	–	2500	100	28.9 ± 0.1
12.5	75	38.6 ± 1.2	–	–	–	1250	100	29.8 ± 0.2
–	–	–	200	100	34.7 ± 0.2	20,000	100	25.7 ± 0.1
–	–	–	100	100	36.2 ± 0.5	10,000	100	26.8 ± 0.2
–	–	–	50	75	37.5 ± 0.8	5000	100	27.9 ± 0.2

*CONC* concentration, *PR* positive rate

**Fig. 2:**
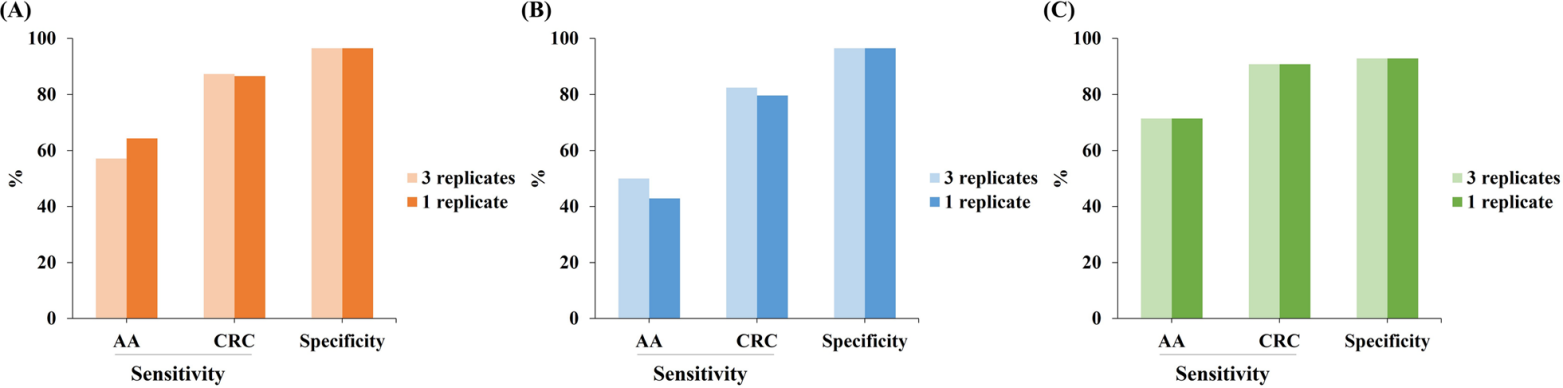
The comparison of sensitivities and specificities for ColoDefense1.0 (3 replicates) and ColoDefense2.0 (1 replicate) in training cohort: **A** *SDC2*, **B** *SEPT9*, **C** ColoDefense

The cut-off value for ColoDefense2.0 was optimized based on the Youden index by using the samples from the training cohort (Additional file 2: Table S1). In order to acquire the best accuracy, we set a minimum sensitivity and specificity of not less than 85%. And the results indicated that when setting the cut-off Ct value of *SEPT9* as 38.0 and that of *SDC2* as 40.0, the ColoDefense2.0 would achieve the best Youden index of 83.7% (Additional file 2: Table S1). Therefore, the subsequent data were analyzed with this cut-off value. In the training cohort, consistent performance of ColoDefense1.0 and ColoDefense2.0 was maintained when reducing the number of replicates from 3 to 1, as shown in Fig. 2 Using *SDC2* alone (Fig. 2A), the sensitivities of AA increased from 57.1 to 64.3%, and that of CRC slightly decreased from 87.3 to 86.6%. Meanwhile, the sensitivities of AA dropped from 50.0 to 42.9%, and that of CRC slightly decreased from 82.4 to 79.6% by using *SEPT9* alone (Fig. 2B). Specificities of both single methylated genes were 96.5% by either ColoDefense test kit, showing an excellent performance on avoiding false positive. As shown in Fig. 2C, the overall performance of ColoDefense1.0 and ColoDefense2.0 was identical in this cohort: the sensitivities for AA and CRC increased to 71.4% and 90.8%, and the specificity was slightly compensated to 92.9%, comparing to those from single methylated genes. Considering the unbalance of age distribution between CRC and control subjects, we excluded the control subjects younger than 40 years old and re-analyzed the specificity, resulting in a specificity of 90.9% (Additional file 2: Table S3).

**Fig. 3:**
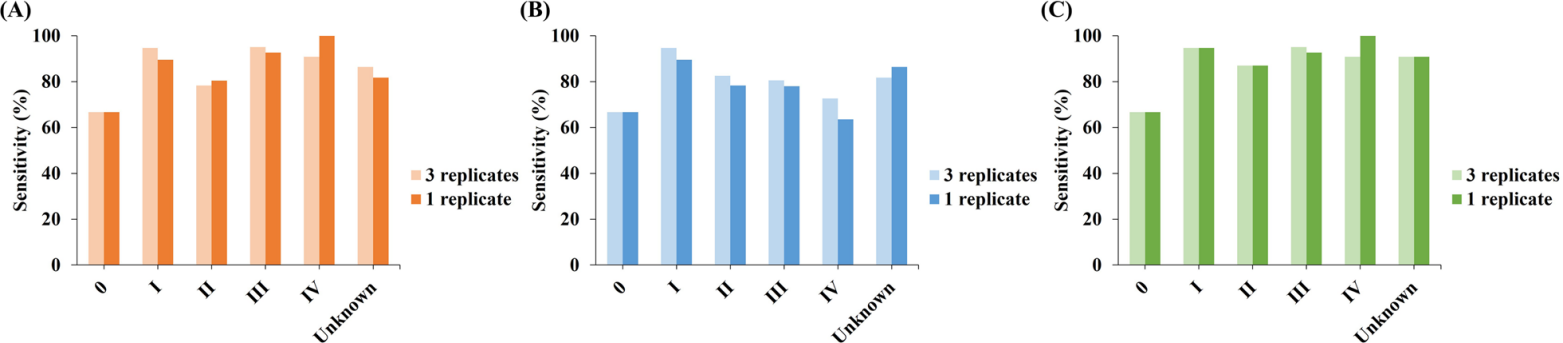
The sensitivities of each stage CRC for ColoDefense1.0 (3 replicates) and ColoDefense2.0 (1 replicate) in training cohort. **A** *SDC2*, **B** *SEPT9*, **C** ColoDefense

For each stage of CRC, the performance of two version of ColoDefense kits were maintained as well (Fig. 3). The sensitivities by *SDC2* in ColoDefense2.0 showed in significant increase of 9.1% for stage IV and minor fluctuations for other stages (Fig. 3A), and those by *SEPT9* showed slight decrease for stage I to IV up to 9.1%, and an increase for unknown stage by 4.6% (Fig. 3B). The overall sensitivities of ColoDefense2.0 (increment from ColoDefense1.0) was 66.7% (0), 94.7% (0), 87.0% (0), 92.7% (− 2.4%), 100% (+ 9.1%), 90.0% (0) for stage 0 to IV and unknown, respectively. Since the number of patients was larger in stage III than that of stage IV, the fluctuation on sensitivities offset each other, leading to the same overall performance of two ColoDefense kits in this cohort.

**Fig. 4:**
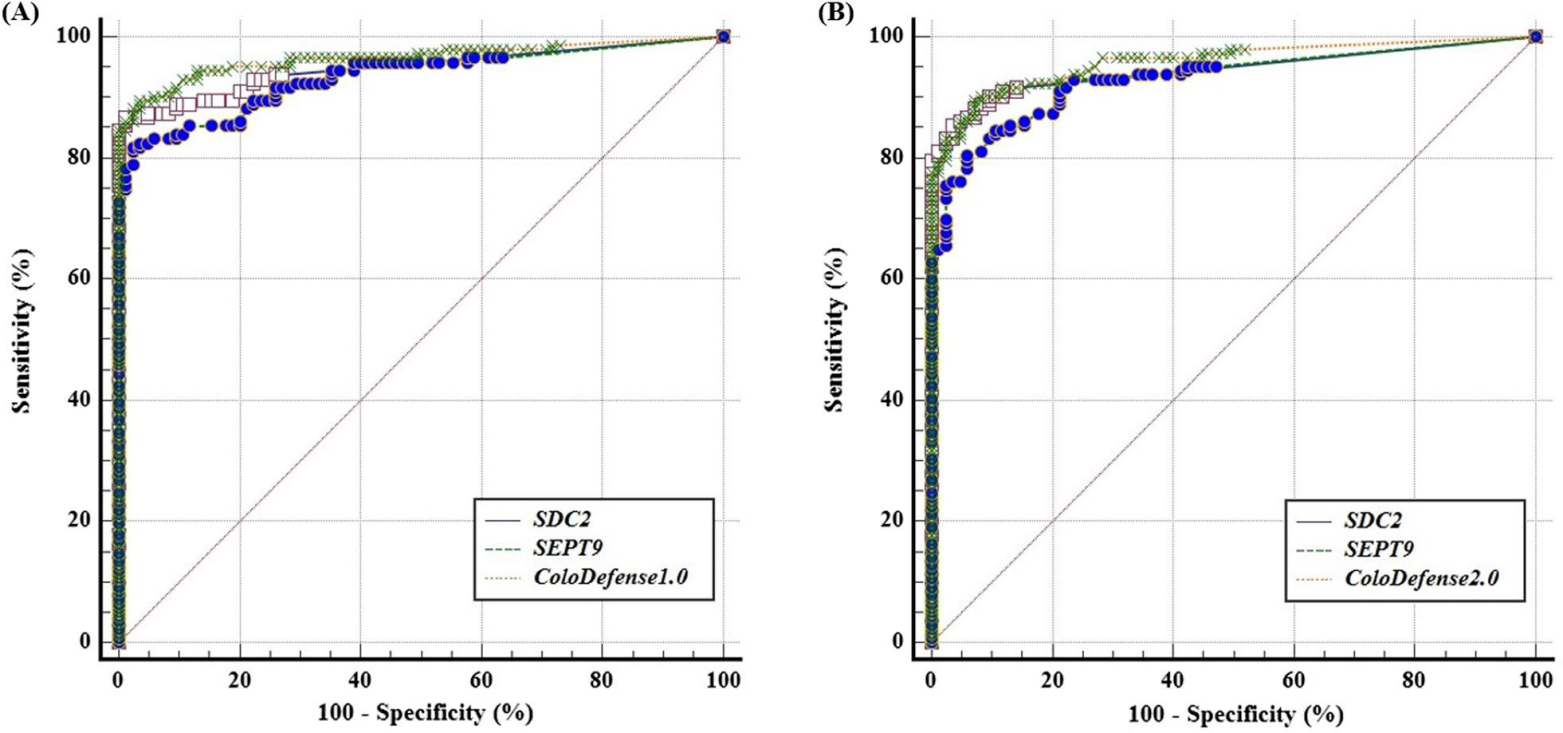
The ROC curves for ColoDefense1.0 **A** and ColoDefense2.0 and **B** in training cohort

Furthermore, AUC of ColoDefense1.0 and ColoDefense2.0 demonstrated no significant differences, as shown in Fig. 4. For ColoDefense1.0 (Fig. 4A), AUCs for *SDC2* and *SEPT9* were 0.948 (95% CI 0.910-0.973) and 0.934 (95% CI 0.893–0.962), respectively, and increased to 0.964 (95% CI 0.931–0.984) if combined. AUCs for ColoDefense2.0 showed no significant change (Fig. 4B), which were 0.945 (95% CI 0.907–0.971), 0.930 (95% CI 0.889–0.960), and 0.959 (95% CI 0.924–0.980) for *SDC2*, *SEPT9* and combined, respectively.

**Fig. 5:**
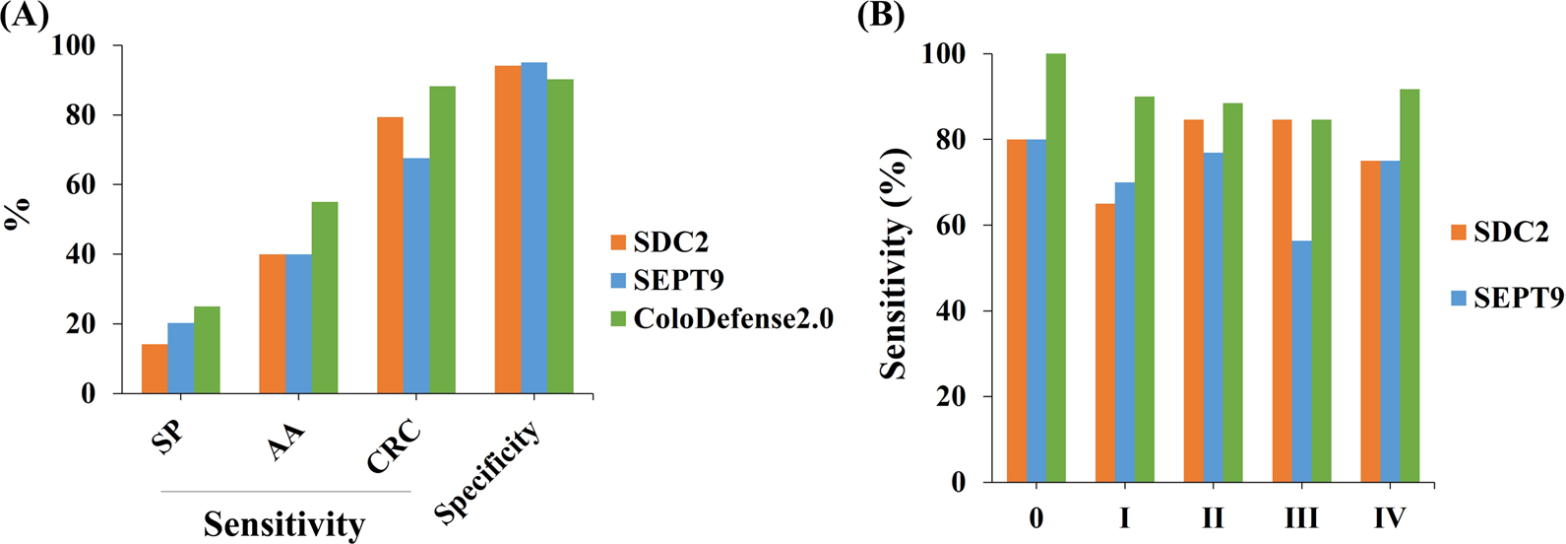
The sensitivities and specificities for ColoDefense2.0 in validation cohort

In the validation cohort, the combination of *SDC2* and *SEPT9* exhibited a significant improvement of sensitivities, especially for precancerous lesions and early stage CRC. As shown in Fig. 5A, comparing to *SDC2* (*SEPT9*) alone, the combined sensitivities of SP, AA and CRC of all stages were increased to 25.0%, 55.0%, 88.2% from 14.1% (20.3%), 40.0% (40.0%), 79.4% (67.6%), respectively. And the combined sensitivities for stage 0 to IV (Fig. 5B) were improved to 100.0%, 90.0%, 88.5%, 84.6% and 91.7% from 80.0% (80.0%), 65.0% (70.0%), 84.6% (76.9%), 84.6% (56.4%) and 75.0% (75.0%), respectively. Moreover, the sensitivity of *SDC2* in detecting CRC were 11.8% higher than that of *SEPT9* alone, and a 3.9% to 4.9% decrease in specificity was observed if two methylated genes were combined. Meanwhile, the specificity in the validation cohort would further decrease to 85.9% if excluding control subjects younger than 40 (Additional file 2: Table S3).

**Fig. 6:**
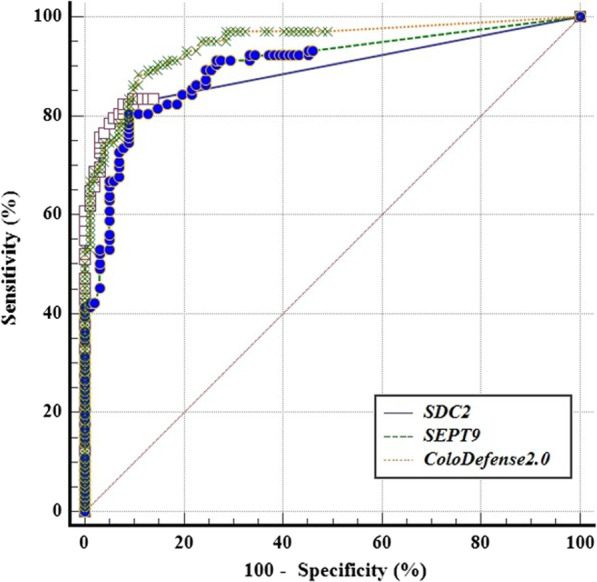
The ROC curves for ColoDefense2.0 in validation cohort

The ROC curves of *SDC2*, *SEPT9* and combined in ColoDefense2.0 in the validation cohort was shown in Fig. 6, AUC values were 0.897 (95% CI 0.847–0.935), 0.898 (95% CI 0.848–0.936) for *SDC2* and *SEPT9*, respectively, and increased to 0.944 (95% CI 0.903–0.971) if combined, all of which were comparable to those in the training cohort. The improvement clearly demonstrated the advantage of optimized multi-target detection.

## Discussion

Screening and early diagnosis of CRC is essential to reduce the incidence and mortality in the long term. A study in 2015 estimated that about 63% of CRC deaths in the US may be attributed to non-screening [15]. Since the screening strategies such as the FOBT and colonoscopy were recommended in 1996–1997 and benefit covered in 2001 in the US, the incidence and mortality have been declining in recent decades [16]. Implementation of screening was considered to attribute to a 50% reduction in CRC incidence and death rate by modeling studies [17]. However, many countries and regions suffered from low participation and compliance rate despite significant benefits from screening, due to limited per capita medical infrastructure and staff, healthcare coverage, awareness and education for patients and providers. Therefore, strategies that provide satisfying accuracy and convenience for both participants and laboratories in relatively low cost and high throughput would be ideal for preliminary screening of CRC. In this study, we optimized a multiplex methylated DNA testing in a single PCR reaction, ColoDefense2.0, providing a potential tool for effective and higher throughput CRC screening.

Multiplex methylated DNA testing for CRC screening has its unique advantage over multiomics tests or single methylation tests. Multiomic tests for CRC screening was developed to achieve improved accuracy for CRC. Cologuard, a stool DNA test including methylated *NDRG4* and *BMP3*, 7 *KRAS* mutations with a hemoglobin immunoassay, reported sensitivities of 42.4% for AA, 69.2% for HGD and 92.3% for all stage CRC, and a specificity of 89.8% from 9989 participants [18]. Such test gained its popularity among insured population in the U.S. due to its effectiveness and convenience. However, high complexity of processing associated with mulitiomics detections has led to a price as high as $649, which could be a major reason to hinder its popularity among uninsured people or in developing countries. Other than multiomic tests, several single-target methylation detection approaches have been launched in succession. However, limitations, such as limited sensitivity for all stages of CRC of *SEPT9* in plasma (68% [12]), decreased sensitivity of stool *SDC2* for late stage CRC (75.6% for stage IV [19]), were observed in these studies. Alternatively, multiplex methylated DNA testing for CRC detection have been developed to achieve improved sensitivity for CRC and for precancerous lesions, with a possible sacrifice of specificity. A recent study on a dual-target stool DNA test (methylated *SDC2* and *TFPI2*) showed a sensitivity of 95.31% for CRC, exceeding the performance of either target alone. Meanwhile, its specificity was reduced from 100% by *SDC2* alone to 96.67% in dual-target test [20]. Similar trends were observed in this study, the sensitivities of CRC increased from 79.4% by *SDC2* and 67.6% by *SEPT9* alone to 88.2% in combined. The sensitivities of SP and AA increased significantly from 14.1% (20.3%) and 40.0% (40.0%) for *SDC2* (*SEPT9*) alone to 25.0% and 55.0% in combination, while the specificity reduced to 90.2% from 94.1% and 95.1% by *SDC2* and *SEPT9* alone. In addition, our multiplex PCR further reduced the cost and increased the detection throughput.

A triplicate PCR reaction was a common strategy to achieve an overall best performance, aiming to achieve a desirable balance between sensitivity and specificity in addition to the cut-off value optimized by Youden index. However, our study demonstrated that such replicates can be optimized and simplified to a single PCR reaction without loss of accuracy. Oh et al*.* reported that a blood-based test detecting *SDC2* methylation in triplicate demonstrated a sensitivity of 87.0% for CRC from 131 patients with a specificity of 95.2% from 125 healthy participants [11]. The blood-based assay Epi proColon was designed to run in triplicate and exhibited a sensitivity of 22% for AA, 68% for all stages of CRC and 64% for stage I-III CRC, with a specificity of 80.0% from a screening population [12]. A triplicate PCR reaction was also applied in a prospective study of methylated *SEPT9* in plasma from 7941 participants, yielding a sensitivity of 48.2% for all stage CRC, and 35.0%, 63.0%, 46.0% and 77.4% for stage I–IV, respectively, with a specificity 91.5% [13]. Our previous study applied and compared 2/3 and 3/3 rules in detecting methylated SFRP2 and *SDC2* in stool to optimize the cut-off values, resulting in sensitivities of 89.1% and 88.5% with specificities of 93.5% and 89.5% in the training and validation cohorts, respectively [14]. In this study, we showed that the sensitivities of ColoDefense2.0 for detecting AA, early stage (0–II) CRC, late stage (III–IV) CRC and unknown stage CRC in the training cohort were 71.4% (10/14), 88.3% (60/68), 94.2% (49/52) and 90.9% (20/22), respectively, with a specificity of 92.9% (79/85), all of which happened to be the same as those of ColoDefense1.0 in triplicate. The sensitivities of ColoDefense2.0 for stage I–IV CRC were 1.7% higher than that from the training cohort in our previous study, and a minor reduction of 0.6% in specificity was also observed. These results implied that the impact of technical replicates in our tests was negligible, and a well-performed strategy was established. More importantly, the detection throughput has been increased by 2 times by reducing the technical triplicates.

Long-term stability of DNA during sampling and transport is the key factor affecting the performance of at-home cancer screening strategy [21]. Our results of stool DNA stability study indicated that the stool DNA collection and transport device used in this study could ensure nearly no DNA degradation in one week at 37 °C, thus we are able to develop a standard work-flow of ColoDefense2.0 and realize nationwide sampling for stool DNA test. Through simplification and/or optimization from the complex multiomics detection to a single multiplex approach, our goal is to reduce the screening cost and push the detection throughput to the limit while ensuring the overall accuracy, so as to encourage as many people as possible to participate in CRC screening. Although the number of participants in this study was limited and only retrospective studies were involved, this study confirmed the feasibility of optimizing the strategies. Further prospective studies of multi-center cohorts in the real-world application still need to be conducted. Meanwhile, there is another limitation of the study: the mean ages of the control subjects were younger than that of the cancer patients, although the adjust specificities in both training and validation cohort only slightly decreased compared with the whole control subjects (Additional file 2: Table S3). We will focus on this point in our future studies.

## Conclusion

By simplifying the procedure of ColoDefense1.0 assay, the ColoDefense2.0 assay maintained its excellent performance on detecting CRC and adenomatous polyps, and increased the PCR reaction throughput in triple, which provided an affordable, accurate and convenient screening tool for CRC.

## Supplementary Information

**Additional file 1:** Supplemental Figure.

**Additional file 2:** Supplemental Tables.

## Data Availability

The datasets used and/or analyzed for the current study are available from the corresponding author upon reasonable request.

## Abbreviations

CRC: Colorectal cancer
AA: Advanced adenomas
SP: Small polyps
FOBT: Fecal occult blood test
FIT: Fecal immunochemical test
cfDNA: Cell-free DNA
PS: Preservative buffer
DS: Deionized water
LoD: Limit of detection
ROC: Receiver operating characteristic
CI: Confidence interval
AUC: Area under the curve
