# Supplementary figures and images for: A simplified multiplex methylated DNA testing for early detection of colorectal cancer in stool DNA
Source: BMC Gastroenterol. 2022 Oct 6;22:428. doi: 10.1186/s12876-022-02512-6 (PMC9540293; doi:10.1186/s12876-022-02512-6)

**Supplemental Figure 1**. The flowchart of this study.


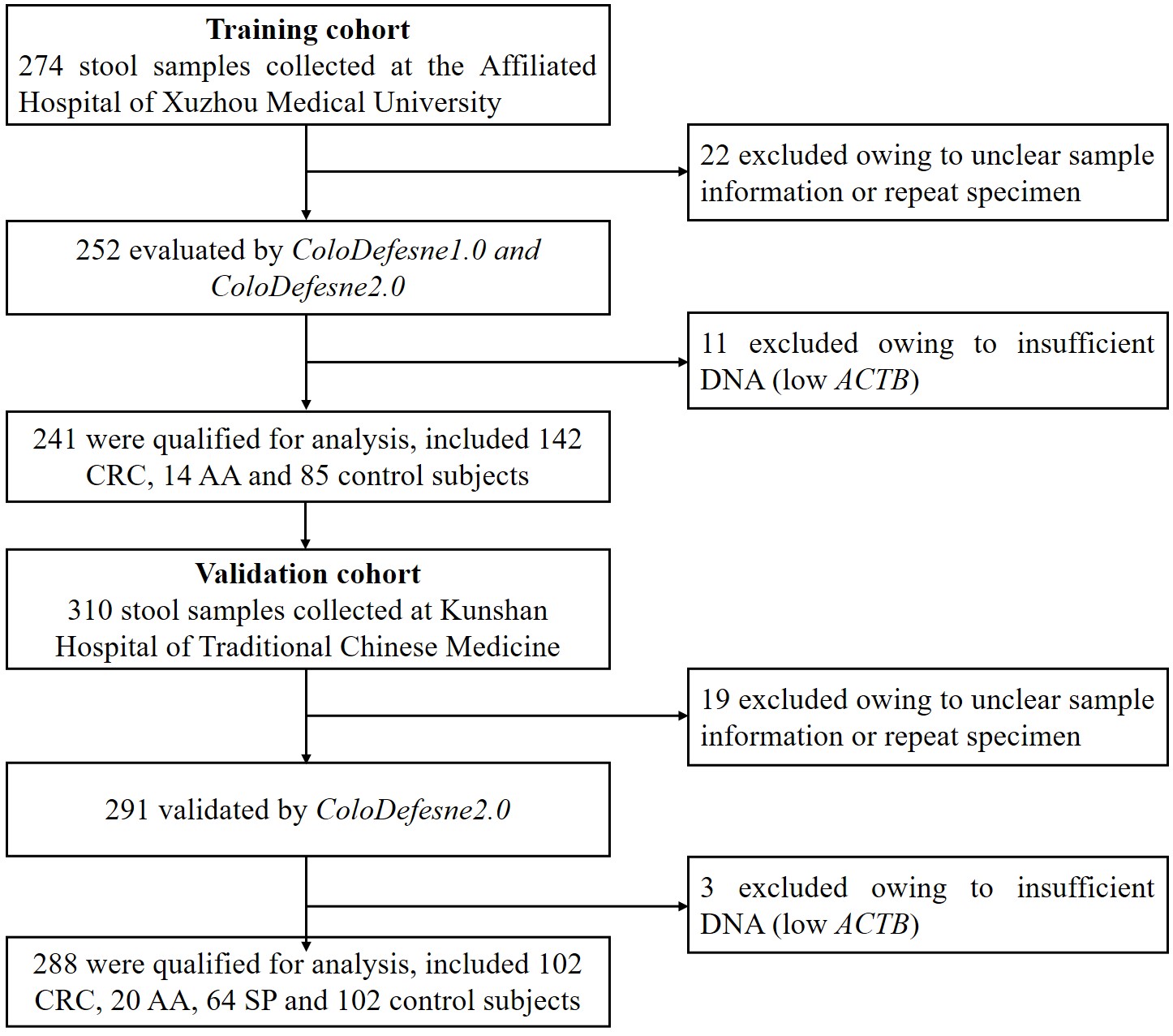

Supplement: Supplementary file 1 — Additional file 1: Supplemental Figure. [file 12876_2022_2512_MOESM1_ESM.docx]
